# Vitamin D is associated with testosterone and hypogonadism in Chinese men: Results from a cross-sectional SPECT-China study

**DOI:** 10.1186/s12958-015-0068-2

**Published:** 2015-07-16

**Authors:** Ningjian Wang, Bing Han, Qin Li, Yi Chen, Yingchao Chen, Fangzhen Xia, Dongping Lin, Michael D. Jensen, Yingli Lu

**Affiliations:** Institute and Department of Endocrinology and Metabolism, Shanghai Ninth People’s Hospital, Shanghai Jiao Tong University School of Medicine, Shanghai, 200011 China; Endocrine Research Unit, Mayo Clinic, Rochester, MN 55905 USA

**Keywords:** Hypogonadism, Vitamin D, Men, Testosterone

## Abstract

**Background:**

To date, no study has explored the association between androgen levels and 25-hydroxyvitamin D (25(OH)D) levels in Chinese men. We aimed to investigate the relationship between 25(OH)D levels and total and free testosterone (T), sex hormone binding globulin (SHBG), estradiol, and hypogonadism in Chinese men.

**Methods:**

Our data, which were based on the population, were collected from 16 sites in East China. There were 2,854 men enrolled in the study, with a mean (SD) age of 53.0 (13.5) years. Hypogonadism was defined as total T <11.3 nmol/L or free T <22.56 pmol/L. The 25(OH)D, follicle-stimulating hormone, luteinizing hormone, total T, estradiol and SHBG were measured using chemiluminescence and free T by enzyme-linked immune-sorbent assay. The associations between 25(OH)D and reproductive hormones and hypogonadism were analyzed using linear regression and binary logistic regression analyses, respectively.

**Results:**

A total of 713 (25.0 %) men had hypogonadism with significantly lower 25(OH)D levels but greater BMI and HOMA-IR. Using linear regression, after fully adjusting for age, residence area, economic status, smoking, BMI, HOMA-IR, diabetes and systolic pressure, 25(OH)D was associated with total T and estradiol (*P* < 0.05). In the logistic regression analyses, increased quartiles of 25(OH)D were associated with significantly decreased odds ratios of hypogonadism (*P* for trend <0.01). This association, which was considerably attenuated by BMI and HOMA-IR, persisted in the fully adjusted model (*P* for trend <0.01) in which for the lowest compared with the highest quartile of 25(OH)D, the odds ratio of hypogonadism was 1.50 (95 % CI, 1.14, 1.97).

**Conclusions:**

A lower vitamin D level was associated with a higher prevalence of hypogonadism in Chinese men. This association might, in part, be explained by adiposity and insulin resistance and warrants additional investigation.

## Background

Vitamin D is a steroid hormone/vitamin that is present in certain foods and may be generated by ultraviolet-B in the skin. Vitamin D is hydroxylated in the liver to 25-hydroxy-vitamin D (25(OH)D), the primary form measured to determine vitamin D status, and can be further hydroxylated to its active form, 1,25-dihydroxy-vitamin D [1]. The classic picture of vitamin D function is the maintenance of bone health and calcium/phosphorous metabolism [2]. However, epidemiological studies have suggested the impact of a pandemic of vitamin D deficiency on non-skeletal functions, including certain cancers, autoimmune diseases, infectious diseases, type 2 diabetes mellitus, neurocognitive disorders, and mortality [3].

Vitamin D receptor (VDR) is present in the testis [4], pituitary gland [5] and hypothalamus [6]. Vitamin D is likely involved in the regulation of gonadal function. Regarding the association between vitamin D and reproduction, replete vitamin D stores predicted reproductive success after in vitro fertilization [7], although excess vitamin levels might have a detrimental impact on the in vitro fertilization outcome [8]. In the USA and Europe, several cross-sectional studies have detected that serum testosterone (T) levels and 25(OH)D levels were positively associated in men [1, 9, 10] and revealed a concordant seasonal variation [1], whereas another study observed no concordant seasonal variation [10]. Therefore, the association of vitamin D with androgens and gonadal function in men has not been fully clarified and has not been reported in the general Chinese population.

We performed a large investigation in 2014, which is referred to as Survey on Prevalence in East China for Metabolic Diseases and Risk Factors (SPECT-China). Using the data from the study, we aimed to determine whether 25(OH)D was associated with the reproductive hormones of the pituitary-testis axis and hypogonadism that was based on total or free T. To the best of our knowledge, the current analyses are the first to focus on several likely explanatory factors contributing to the relationship of 25(OH)D and hypogonadism, including adiposity, insulin resistance and other metabolic factors.

## Methods

### Study population

SPECT-China is a population-based cross-sectional survey on the prevalence of metabolic diseases and risk factors in East China from February to June 2014. The registration number is ChiCTR-ECS-14005052 (www.chictr.org). We used a stratified cluster sampling method. The first sampling level was based on rural and urban areas and the second sampling level was based on an area’s economic development. This study was performed in Shanghai, Jiangxi Province and Zhejiang Province (three sites in urban areas of Shanghai, one site in an urban area of Jiangxi Province, three sites in rural areas in Shanghai, three sites in rural areas in Zhejiang and six sites in rural areas in Jiangxi Province) [11]. The inclusion criteria were as follows: adults aged 18 years and older; Chinese citizens; and living in their current residences for 6 months or longer. Those who had severe communication problems or acute illnesses or were unwilling to participate were excluded from the study.

A total of 7200 people participated in this investigation. The participants who had missing laboratory results (*n* =183) and missing questionnaire data (*n* =112) and were younger than 18 years of age (*n* =6) were excluded from the study; a total of 6,899 subjects were enrolled in the SPECT-China study [11]. Among them, there were 2940 men who did not receive vitamin D and testosterone supplementation. Men with missing values of 25(OH)D (*n* =1), total testosterone (*n* =7) and free testosterone (*n* =78) were excluded from the study. Finally, this study included a total number of 2854 men with a mean (SD) age of 53 (13) years. The study protocol was approved by the Ethics Committee of Shanghai Ninth People’s Hospital, Shanghai Jiao Tong University School of Medicine (approval number 2013 (86)). The research was conducted according to the Declaration of Helsinki. All of the participants provided written informed consent before data collection.

### Measurements

In every site, the same trained staff group completed the questionnaire including information on demographic characteristics, medical history and lifestyle risk factors, and they collected anthropometric data. Current smoking was defined as having smoked at least 100 cigarettes in one’s lifetime and currently smoking cigarettes [12]. Body weight, height, and blood pressure were measured using standard methods, as previously described [12]. Body mass index (BMI) was calculated as weight in kilograms divided by height in meters squared.

The participants fasted for 8 h before the study. Fasting blood samples were drawn between 0700 h and 1000 h. The blood samples for the plasma glucose test were collected in vacuum tubes with anticoagulant sodium fluoride and centrifuged on the spot within 1 h after collection. The blood samples were stored at −20 °C and were shipped by air in dry ice within 2 to 4 h of collection to a central laboratory, which is certified by the College of American Pathologists. Glycated hemoglobin (HbA1c) was assessed using high-performance liquid chromatography (MQ-2000PT, China). The plasma glucose was measured using Beckman Coulter AU680 (Germany). Insulin was detected by chemiluminescence (Abbott i2000 SR, USA). The 25(OH)D, total T, estradiol (E2), follicle-stimulating hormone (FSH) and luteinizing hormone (LH) were measured using the immulite 2000 platform chemiluminescence immunoassays (Siemens, Germany) and sex hormone binding globulin (SHBG) by electrochemiluminescence (Roche Cobas E601, Switzerland). Free T was detected using enzyme-linked immunosorbent assay (Bio-Tek ELx808, USA). The minimal detectable limit for hormones was as follows: 0.7 nmol/L (total T), 73.4 pmol/L (E2), and 0.1 IU/L (FSH and LH). The inter-assay coefficients of variation were as follows: 15 % (free T), 6.6 % (total T), 7.5 % (E2), 7 % (SHBG), 4.5 % (FSH) and 6.0 % (LH). The intra-assay coefficients of variation were as follows: 15 % (free T), 5.7 % (total T), 7 % (SHBG), 6.2 % (E2), 3.8 % (FSH) and 4.9 % (LH). Insulin resistance was estimated using the homeostatic model assessment (HOMA-IR) index: [fasting insulin (mIU/L) × fasting glucose (mmol/L)]/22.5.

### Definition of variables and outcomes

Hypogonadism was defined as total T <11.3 nmol/L [13] or free T <22.56 pmol/L [14] in men. Based on the American Diabetes Association 2014 criteria, diabetes was defined as a previous diagnosis by healthcare professionals, fasting plasma glucose ≥7.0 mmol/L, or HbA1c ≥6.5 %. We considered the residence area as a covariate because in China the prevalence of hypogonadism in rural and urban areas may be different [15]. The economic development status was measured by the average gross domestic product per capita of the entire nation (6807 US dollars, from the World Bank) in 2013 as the cutoff point for each site.

### Statistical analysis

We performed survey analyses using IBM SPSS Statistics, Version 22 (IBM Corporation, Armonk, NY, USA). All of the analyses were two-sided. *P* < 0.05 was considered to be statistically significant. All reported *P* values were 2-sided. The general characteristics are summarized as the mean (SD) values for continuous variables or as a number with proportion for categorical variables. To test for differences in characteristics between the participants with and without hypogonadism, and among 25(OH)D quartiles, Kruskal-Wallis and one-way ANOVA were used for non-normally and normally distributed continuous data, respectively, and the Pearson *χ*^2^ test was used for categorical variables. The missing values of total T (*n* = 3) and E2 (*n* = 779) under the minimal detectable limit were replaced by the mean values that were 0.35 nmol/L and 36.7 pmol/L between 0 and the minimal detectable limit.

The association of 25(OH)D (dependent variable) with total T, free T, E2, SHBG, FSH and LH (independent variables) was assessed using linear regression. Model 1 was unadjusted. Model 2 was adjusted for age, residence area, economic development and current smoker. Model 3 included terms for model 2, BMI, log (HOMA-IR), diabetes and systolic pressure. Because free T, SHBG, FSH, LH and HOMA-IR were non-normally distributed, they were transformed by natural logarithm. The results were expressed as unstandardized coefficients (B) and 95 % confidence intervals (CIs).

The 25(OH)D was divided into quartiles, with the first quartile representing the lowest one and the fourth quartile representing the highest. Odds ratio (OR) and 95 % CI were calculated using logistic regression to determine the risk of hypogonadism for each quartile of 25(OH)D, with the highest quartile as the reference. Model 1 was adjusted for age, residence area, economic status and current smoker. Model 2 included terms for model 1, BMI and HOMA-IR. Model 3 included terms for model 2, diabetes and systolic pressure.

We tested the interaction effect between 25(OH)D and residence area, economic status, HbA1c, BMI and HOMA-IR by adding a multiplicative factor in the logistic regression model.

## Results

### Characteristics of the study population

General demographic and laboratory characteristics of the study population are summarized in Table 1. Seven hundred thirteen (25.0 %) participants had hypogonadism. Among these characteristics, 501 men were diagnosed with hypogonadism by total T, 334 men were diagnosed by free T and 122 men were diagnosed by both total and free T. Compared to men without hypogonadism, the men with hypogonadism were older and had significantly lower 25(OH)D levels, higher fasting insulin levels, HOMA-IR, BMI and systolic pressure as well as a higher prevalence of diabetes. With regard to hormones in the pituitary-testis axis, the men with hypogonadism had significantly lower total T, free T, E2, SHBG and higher FSH levels.

**Table 1:** General characteristics of the participants

	Men without hypogonadism	Men with hypogonadism	*P*
*N*	2141	713	
Age, yr	52 (14)	55 (13)	<0.01
Metabolic factors			
25(OH)D, nmol/L	43.7 (11.7)	42.6 (11.8)	<0.01
HbA1c, %	5.4 (0.8)	5.6 (1.1)	<0.01
Fasting glucose, mmol/L	5.58 (1.24)	5.97 (1.76)	<0.01
Fasting insulin, pmol/L	35.6 (32.5)	48.9 (60.0)	<0.01
HOMA-IR	1.31 (1.46)	1.96 (2.83)	<0.01
Systolic pressure, mmHg	131 (20)	134 (20)	<0.01
BMI, kg/m^2^	24.2 (3.2)	25.4 (3.6)	<0.01
Diabetes, %	10.7	19.9	<0.01
Sex-related hormones			
Total T, nmol/L	17.8 (5.3)	11.2 (4.3)	<0.01
Free T, pmol/L	54.0 (26.9)	33.0 (27.1)	<0.01
E2, pmol/L	112.8 (64.5)	90.8 (55.9)	<0.01
SHBG, nmol/L	48.7 (24.9)	38.1 (24.5)	<0.01
FSH, IU/L	8.4 (6.1)	11.3 (14.3)	<0.01
LH, IU/L	5.3 (3.3)	6.0 (5.8)	0.05
Rural/urban residence, %	56.0/44/0	58.9/41/1	0.18
Economic status, {low/high, %}	28.7/71.3	22.9/77.1	<0.01
Current smoker, %	48.9	44.8	0.07

HOMA-IR, homeostasis model assessment-insulin resistance; E2, estradiol; SHBG, sex hormone binding globulin; FSH, follicle-stimulating hormone; HbA1c, glycated hemoglobin; LH, luteinizing hormone

The data are summarized as the mean (standard deviation) for continuous variables, or as number with proportion for categorical variables. The Mann–Whitney *U* test was used for non-normally distributed continuous variables, and the Pearson *χ* ^2^ test was used for dichotomous variables

Table 2 shows the characteristics of the study population according to serum 25(OH)D quartiles. The quartile ranges of 25(OH)D were ≤35.36 nmol/L, 35.37-41.32 nmol/L, 41.33-48.81 nmol/L and ≥48.82 nmol/L. Compared with men in the highest quartile, the men in the lowest quartile were younger and were more likely to live in an urban area and a rapid economic development area; however, they had a higher prevalence of hypogonadism and diabetes. These men also had significantly lower total T, E2, SHBG, FSH and LH levels but higher free T levels.

**Table 2:** Characteristics of the participants by quartiles of 25(OH)D

	Q1	Q2	Q3	Q4	
25(OH)D, nmol/L	≤35.36	35.37-41.32	41.33-48.81	≥48.82	*P*
*N*	714	715	712	713	
Age, yr	50 (14)	51 (13)	53 (13)	59 (13)	<0.01
Hypogonadism, %	28.6	26.0	22.8	22.6	<0.05
Metabolic factors					
HbA1c, %	5.5 (1.0)	5.4 (0.9)	5.4 (0.9)	5.5 (0.9)	<0.05
Fasting glucose, mmol/L	5.68 (1.51)	5.63 (1.45)	5.68 (1.31)	5.71 (1.34)	<0.05
Fasting insulin, pmol/L	42.2 (48.5)	40.8 (38.0)	37.3 (32.5)	35.3 (45.1)	<0.01
HOMA-IR	1.60 (2.33)	1.53 (1.81)	1.41 (1.50)	1.34 (1.94)	<0.01
Systolic pressure, mmHg	132 (19)	131 (20)	130 (19)	135 (21)	<0.01
BMI, kg/m^2^	24.3 (3.3)	24.9 (3.5)	24.6 (3.1)	24.2 (3.2)	<0.01
Diabetes, %	14.7	11.5	13.4	12.5	0.31
Sex-related hormones					
Total T, nmol/L	15.5 (5.7)	15.8 (5.7)	16.2 (5.7)	17.1 (6.1)	<0.01
Free T, pmol/L	50.6 (29.0)	51.1 (33.9)	49.0 (26.4)	44.4 (23.0)	<0.01
E2, pmol/L	99.9 (59.2)	101.5 (59.8)	106.0 (61.3)	121.9 (69.5)	<0.01
SHBG, nmol/L	42.4 (23.4)	41.8 (22.4)	45.2 (23.7)	54.8 (28.7)	<0.01
FSH, IU/L	8.1 (7.5)	8.2 (6.6)	8.6 (6.8)	11.4 (13.0)	<0.01
LH, IU/L	5.0 (3.9)	5.1 (3.5)	5.2 (2.8)	6.6 (5.4)	<0.01
Rural/urban residence, %	51.1/48.9	49.0/51.0	53.5/46.5	73.2/26.8	<0.01
Economic status, {low/high, %}	17.9/82.1	27.6/72.4	30.8/69.2	32.8/67.2	<0.01
Current smoker, %	50.7	50.4	46.2	44.0	<0.05

HOMA-IR, homeostasis model assessment-insulin resistance; T, testosterone; E2, estradiol; SHBG, sex hormone binding globulin; FSH, follicle-stimulating hormone; HbA1c, glycated hemoglobin; LH, luteinizing hormone

The data are summarized as the mean (standard deviation) for continuous variables, or as number with proportion for categorical variables. The Kruskal-Wallis test was used for non-normally distributed continuous variables, and the Pearson *χ* ^2^ test was used for dichotomous variables

### Association of 25(OH)D with reproductive hormones

Table 3 summarizes the results of the linear regression models studying the association of 25(OH)D with total T, free T, E2, SHBG, LH and FSH. In the unadjusted models, the higher levels of 25(OH)D were associated with lower log (free T) (= − 0.002), and higher total T (*B =* 0.056), E2 (*B =* 0.866), log SHBG (*B =* 0.004), log LH (*B =* 0.004) and log FSH (*B =* 0.005) (all *P* < 0.001). After adjustment for age, residence area, economic status and current smoker, the association of 25(OH)D with log (free T) weakened further to the extent that it was no longer significant. After additional adjustments for BMI, HOMA-IR, diabetes and systolic pressure, only total T (*B =* 0.023) and E2 (*B =* 0.238) were still associated with 25(OH)D (both *P* < 0.05).

**Table 3:** Association of 25(OH)D with sex-related hormones

	25(OH)D, nmol/L		
Dependent variables	Model 1	Model 2	Model 3
Total T, nmol/L	0.056 (0.038, 0.074)***	0.031 (0.011, 0.051)**	0.023 (0.004, 0.042)*
Log (free T), pmol/L	−0.002 (−0.003, −0.001)***	−0.001 (−0.001, 0.000)	−0.001 (−0.001, 0.000)
E2, pmol/L	0.866 (0.671, 1.061)***	0.264 (0.054, 0.475)*	0.238 (0.023, 0.452)*
Log SHBG, nmol/L	0.004 (0.004, 0.005)***	0.001 (0.000, 0.002)**	0.001 (0.000, 0.001)
Log LH, IU/L	0.004 (0.003, 0.004)***	0.001 (0.000, 0.001)*	0.001 (0.000, 0.001)
Log FSH, IU/L	0.005 (0.004, 0.006)***	0.001 (0.000, 0.002)*	0.001 (0.000, 0.002)

The data are expressed as *B* coefficient (95 % confidence interval)

T, testosterone; E2, estradiol; SHBG, sex hormone binding globulin

Model 1 was unadjusted. Model 2 included terms for age, residence area, economic status and current smoker. Model 3 included terms for model 2, BMI, log (HOMA-IR), diabetes and systolic pressure. To obtain the average percentage change in free T, SHBG, LH or FSH from log hormone = 100 × (exp(*B* coefficient) - 1)

*P < 0.05, **P < 0.01, ***P < 0.001. Linear regression analysis was used

### Association of 25(OH)D with hypogonadism

Table 4 demonstrates the results of the binary logistic regression measuring the association of 25(OH)D status with hypogonadism. In all of the models, the odds ratios for hypogonadism decreased across 25(OH)D quartiles (all *P* for trend <0.01). Compared with men in the highest quartile of 25(OH)D (Table 4, model 1), the odds ratio of hypogonadism in men in the lowest quartile of 25(OH)D were 1.54 (95 % CI, 1.19, 2.01; *P* < 0.01). After adjustments for BMI and HOMA-IR, the association was so weakened (Table 4, model 2) that it was no longer significant in Q2. Additional adjustment for diabetes and systolic blood pressure did not weaken this association (Table 4, model 3). No interaction was observed between 25(OH)D, and diabetes, BMI and HOMA-IR.

**Table 4:** Association of 25(OH)D with hypogonadism

25(OH)D, nmol/L	Model 1	Model 2	Model 3
Q1 (≤35.36)	1.54 (1.19, 2.01)	1.49 (1.14, 1.95)	1.50 (1.14, 1.97)
Q2 (35.37-41.32)	1.38 (1.06, 1.80)	1.26 (0.96, 1.66)	1.27 (0.97, 1.67)
Q3 (41.33-48.81)	1.07 (0.82, 1.40)	1.04 (0.79, 1.36)	1.03 (0.79, 1.36)
Q4 (≥48.82)	1.00	1.00	1.00
*P* value for trend	<0.01	<0.01	<0.01

The data are expressed as odds ratio (95 % CI) unless otherwise indicated. Logistic regression analysis was used

Model 1 included terms for age, residence area, economic status, and current smoker. Model 2 included terms for model 1, BMI, and HOMA-IR. Model 3 included terms for model 2, diabetes, and systolic pressure. No interaction was found between 25(OH)D, and BMI and HOMA-IR

## Discussion

In this population-based study of middle-aged and older Chinese men, we observed that lower 25(OH)D level was significantly associated with lower total T, E2, SHBG, LH and FSH levels after adjusting for age, residence area, economic status and current smoker. Additional adjustments for BMI, HOMA-IR, diabetes and systolic pressure revealed that total T and E2 were still associated with 25(OH)D. The fully adjusted odds ratio of hypogonadism increased by 50 % for the lowest quartile compared with the highest quartile of 25(OH)D. Adiposity and insulin resistance may, in part, explain this association. To the best of our knowledge, this study is the first to detect the association between 25(OH)D status and hypogonadism in Chinese men and confirms that this relationship is present in a large population.

Vitamin D deficiency, which affects not only the musculoskeletal system but also a number of acute and chronic diseases, is currently a pandemic health problem. In 1711 healthy Caucasian adults, the mean serum vitamin D concentration was 64.4 nmol/L in men [16]. In 136 healthy Japanese males, the median 25(OH)D concentration was 36.5 nmol/L and approximately 75 % of the subjects had 25(OH)D-deficiency (25(OH)D <50 nmol/L) [17]. In our participants, the fourth quartile of 25(OH)D was 48.82 nmol/L, so at least 75 % had suboptimal 25(OH)D. Interestingly, serum 25(OH)D was higher in older men than in younger men. This finding may be due to more indoor and sedentary activities in the young, whereas the older, retired men may engage in more outdoor activities that result in more sun exposure; this result warrants additional investigation.

Our results demonstrating an association of vitamin D status and testosterone in men are also consistent with outcomes in animal studies. VDR knockout mutant mice showed gonadal insufficiencies. High LH and FSH levels in the male mice indicated hypergonadotropic hypogonadism [18]. Another mouse study reported a tendency towards low testosterone/LH ratio and Leydig cell hyperplasia in VDR null mice [19]. The serum testosterone levels could increase to normal values in vitamin D-deficient rats replete with vitamin D [20]. However, controversy exists regarding to what extent vitamin D is important for sex hormone production. Blomberg Jensen et al. found that testosterone and LH serum levels were not significantly different between wild­type and VDR­null mice [19]. Additionally, vitamin D may also regulate reproductive function. VDR knockout mice had decreased sperm count, reduced sperm motility, and histological abnormality of the testis [18]. Pilz et al. [21] reported that vitamin D supplementation increases testosterone levels in non-diabetic subjects, although Jorde et al. [22] could not detect an effect of vitamin D supplements on serum testosterone concentrations in healthy men.

Our results are consistent with three previous population-based reports [1, 10, 14]. The data from the European Male Ageing Study [9] indicated that 25(OH)D is positively associated with total T. However, after adjusting for health and lifestyle factors, no significant associations were observed. This study recruited non-institutionalized, primarily Caucasian men, and the data may, therefore, not be generalizable to other groups. The association of 25(OH)D with free T varied [1, 9, 10, 14]. Because of the strong inter- and intra-assay coefficients of variation of free T measurements, the associations of 25(OH)D with free T should be viewed with caution [10].

In our study, hypogonadism was defined as total T <11.3 nmol/L or free T < 22.56 pmol/L, and the prevalence of hypogonadism was 25.0 % among our participants with a mean age of 53 years. In the Hypogonadism in Males study in the USA, the prevalence of hypogonadism was 38.7 % in men aged ≥45 years [23] and in Taiwanese men aged >40 years; 24.1 % had androgen deficiency [24], both of which were based on total T. Our prevalence seemed to be comparable with that observed in Taiwanese men. Such a high prevalence of hypogonadism was found to be associated with pandemic vitamin D deficiency in our study. Our results included the evidence from a new ethnic group reported in previous studies [1, 9, 10, 14]. We observed that adjustments for BMI and HOMA-IR attenuated the association between vitamin D and hypogonadism, indicating that this association was in part explained by adiposity and insulin resistance. The association between obesity and hypogonadism has been well researched [25]. Previous studies have reported that obesity is associated with low serum 25(OH)D levels [26]. Vitamin D may regulate adipose tissue [27]. VDR is observed in human adipocytes [28], and vitamin D deficiency is capable of up-regulating parathyroid hormone, thereby increasing free intracellular calcium in adipocytes, which blunts the lipolytic response to catecholamines and enhances lipogenesis [29]. Thus, this association may, in part, be mediated through adiposity.

Our study had several strengths. First, regarding the significance, this study is the first to detect the association between 25(OH)D status and hypogonadism in general Chinese men, the largest population group in the world, thereby adding new evidence to previous research. Second, this study has strong quality control; the anthropometric measurements and questionnaires were all completed by the same trained research group, and all of the biomedical measurements were performed in one laboratory certified by the College of American Pathologists. Third, our results are more reflective because the SPECT-China study was performed in a general population as opposed to a clinic-based population. However, our study has several limitations. First, due to the cross-sectional nature of the study, we cannot draw a causal relationship between 25(OH)D status and hypogonadism. Second, this study recruited primarily Han Chinese men and the data cannot be generalizable to other ethnic groups. Third, we measured reproductive hormones and vitamin D once, indicating that we could not perform the seasonal variation analysis.

## Conclusions

Vitamin D deficiency is pandemic in Chinese men. Lower vitamin D levels were associated with a higher prevalence of hypogonadism in Chinese men. This association was, in part, explained by adiposity and insulin resistance, which warrants additional investigation.

## Abbreviations

25(OH)D: 25-hydroxy-vitamin D
BMI: Body mass index
CI: Confidence interval
E2: Estradiol
FSH: Follicle-stimulating hormone
HbA1c: Glycated hemoglobin
HOMA-IR: Homeostatic model assessment-insulin resistance
LH: Luteinizing hormone
OR: Odds ratio
SHBG: Sex hormone binding globulin
VDR: Vitamin D receptor
